# P-742. Preclinical evaluation of a novel single-visit point-of-care multiplex diagnostic test *Chlamydia trachomatis* and *Neisseria gonorrhoeae*

**DOI:** 10.1093/ofid/ofaf695.953

**Published:** 2026-01-11

**Authors:** Amangeldy Urazov, Sara Foreman, Peter Rahfeld, Violette Defourt

**Affiliations:** Rapidemic, Leiden, Zuid-Holland, Netherlands; Rapidemic, Leiden, Zuid-Holland, Netherlands; Rapidemic, Leiden, Zuid-Holland, Netherlands; Rapidemic, Leiden, Zuid-Holland, Netherlands

## Abstract

**Background:**

According to the WHO, an estimated 128,5M *Chlamydia trachomatis* and 86,4M *Neisseria gonorrhea* (NG) infections are acquired every single year. The golden standard for diagnosis are nucleic acids amplification tests, where a sample is analyzed in a central diagnosis laboratory. In many settings, this is suboptimal as it is logistically demanding, requires trained personnel, and has a slow turnover (< 7 d). This has consequences for clinicians and individual patients. The WHO has been advocating for the implementation of point-of-care tests for these infections to improve diagnosis and treatment, and recent developments in the field have shown promising potential to tackle the rise in CT/NG infections. However, current available technologies are either inaccurate, complex, slow or unaffordable.

Rapidemic develops a 15-minute portable molecular test for the diagnosis of CT/NG from vaginal swabs and urine. The device is a disposable, equipment-free test cassette, which allows users to test in clinics and at-home. The test is highly user-friendly and consists of 4 consecutive steps: sample collection, lysis, amplification and read-out using a visual lateral flow strip.

**Methods:**

The aim of this project was to determine preclinical performance of the assay. To do so, the lysis capacity of Rapidemic’s innovative room-temperature and instant lysis buffer was first determined on NG and CT bacteria. Secondly, the patient matrix tolerance was assessed in urine and vaginal swabs. Lastly, the performance of the test was determined in a head-to-head comparison with the QIAGEN NeuMoDx CT/NG test on fresh patient urine and vaginal swabs obtained in a local STI clinic.

**Results:**

The outcome of this project demonstrated a high tolerance (< 10-40%) for raw sample input in the assay. Viability data on different formulations demonstrated that the lysis buffer has the capability to lyse CT and NG bacteria instantly at room temperature. Finally, the results demonstrate a 100% NPV and PPV with the comparator on urine and vaginal swabs (n= 23 and 10).

**Conclusion:**

The Rapidemic test can lyse and detect CT and NG bacteria in urine and swab material in under 15 minutes. This decentralized test fits the WHO’s ASSURED criteria and could be a promising tool for implementation in primary healthcare settings around the world.

**Disclosures:**

Sara Foreman, n/a, Rapidemic: Stocks/Bonds (Private Company) Peter Rahfeld, n/a, Rapidemic: Stocks/Bonds (Private Company) Violette Defourt, n/a, Rapidemic: Ownership Interest|Rapidemic: Ownership Interest|Rapidemic: Stocks/Bonds (Private Company)

